# Primum succurrere; first hasten to help

**DOI:** 10.1093/oncolo/oyae368

**Published:** 2025-04-11

**Authors:** Dan L Longo, James O Armitage

**Affiliations:** Department of Medicine, Brigham and Women’s Hospital and Harvard Medical School, Boston, MA 02115, United States; Department of Medicine, University of Nebraska Medical Center, Omaha, NE 68198, United States

**Keywords:** primum non nocere, primum succurere, physician’s mission

One of the first things we are taught in medical school is the well-known injunction “primum non nocere” or “first do no harm.” While this aphorism is entrenched in medical lore, this phrase is not found in the Hippocratic Oath.^1^ It was added as an interpretation subsequently. Though the first mention of this exegesis of the Oath is debated, it may have been a word-of-mouth spread of the teachings of the Parisian pathologist, Auguste Francois Chomel, Laennec’s successor at the Faculte de Paris.^2^ Given the nature of medical intervention until the 20th century development of clinical investigation, it was reasonable to prioritize not doing harm with interventions not proven effective. With the paucity of tools available, many if not most medical interventions ended with the patient worse off than at the start. While we would likely all agree that doing harm to patients should be avoided whenever possible, the only way to be sure one has done no harm is to do nothing, an approach that is clearly not part of the physician’s mission.

The problem with this passive approach to patient care is that almost all medical interventions (including surgery, medications, diagnostic studies, even physical diagnosis) are associated with some degree of risk. Many times the risks are trivial, but not always. Both of us are oncologists who primarily care for patients with lymphoma. Our patients have diseases that are almost always fatal if not effectively treated. For aggressive lymphomas, this requires therapy that is curative, while in some indolent lymphomas, treatment that can control the disease for long periods of time with the patient remaining asymptomatic is utilized.

The treatments that can achieve these results (eg, combination chemotherapy, bone marrow transplantation, new immunotherapies, targeted therapies, etc.) were developed in clinical studies by patients and physicians who were brave enough to take risks of serious and sometimes fatal toxicity hoping for individual benefit, but also to advance the care of future patients in the same situation. Even today, the treatments administered to cure patients of lymphoma and other cancers have real risks of short- and long-term toxicity including death. And, of course, ours is not the only specialty that has to address these issues.

Shared decision-making is an important part of being an effective physician. A physician should never lose sight of who works for whom. No patient should receive any treatment—much less a dangerous one—unless they understand the potential risks and benefits and want to go forward. We believe that most patients are willing to accept risks of harm rather than pass up the chance to cure or extend survival in an otherwise fatal disease. This is true of both short- and long-term toxicity. At a national meeting years ago Dr Vincent DeVita, who developed the first curative therapy for a solid tumor, responded to criticism that the MOPP regimen was causing acute myeloid leukemia in about 5% of long survivors of Hodgkin lymphoma. He responded that “it is better to die of AML tomorrow than Hodgkin’s disease today.”

It is important to remember that treatments for cancer are often given at the maximum tolerated dose. However, the maximum tolerated dose is really variable and dependent on the assessment of the risk/benefit ratio in a particular setting. Is the goal of treatment cure or palliation? What is the patient’s physiologic reserve, the capacity to bounce back from a treatment-induced adverse effect? Most of us would set the maximum tolerated dose lower for a patient with metastatic pancreatic cancer than for a patient with acute leukemia or aggressive lymphoma. If we believe that a regimen has no potential for cure, we are much less likely to ask the patient to put up with serious toxicity. However, we routinely administer treatments with life threatening toxicities if there is a reasonable chance for cure and if the toxicities are usually reversible.

Primum non nocere may not merit the obeisance with which it has been adorned. Indeed, many things in the Oath are readily and appropriately ignored (as cited by Abraham Verghese, it prohibits surgery to remove bladder stones^3^) and primum non nocere is not even in it.^1^ Perhaps, the saying “first do no harm” is anachronistic for much of modern medicine in general and for oncology in particular. Today, we do not just offer nostrums and passively chronicle the natural history of disease. We intervene, we invade, and we alter natural history. Perhaps, a better motto for modern physicians than primum non nocere would be “primum succurrere,” meaning first hasten to help your patient. To do the best you can to treat serious illnesses, risks of harm are omnipresent. It is the risk to benefit ratio that is our guiding light. If you would not ever risk doing harm you will miss the chance of doing a lot of good. Indeed, a careful reading of the Hippocratic Oath is consonant with this approach. According to a translation from Greek by Francis Adams^4^ from 1849 that is quoted by Britannica (https://www.britannica.com/topic/Hippocratic-oath), the following appears: “I will follow that system of regimen which, according to my ability and judgment, I consider for the benefit of my patients, and abstain from whatever is deleterious and mischievous.” It would seem that even the original document prioritizes beneficence over doing no harm.
